# Cognitive impairment or delusional disorder? A case report

**DOI:** 10.1192/j.eurpsy.2025.1751

**Published:** 2025-08-26

**Authors:** B. Rodríguez Rodríguez, N. Navarro Barriga, C. Rodríguez Valbuena, M. Fernández Lozano, J. C. Fiorini Talavera, G. Guerra Valera, A. Aparicio Parras, P. Marínez Gimeno, M. A. Andreo Vidal, M. Calvo Valcárcel, M. P. Pando Fernández, G. Lorenzo Chapatte, M. Ríos Vaquero, A. Monllor Lazarraga, L. Rojas Vázquez, F. J. González Zapatero, L. Del Canto Martínez, M. B. Arribas Simón

**Affiliations:** 1 Psiquiatría, Hospital clínico universitario de Valladolid, Valladolid, Spain

## Abstract

**Introduction:**

Delusions and hallucinations can appear in various psychiatric and neurological pathologies. When these psychotic symptoms are of late onset, in geriatric age, it may be necessary to make a differential diagnosis between dementia or other psychiatric disorders.

**Objectives:**

To describe the differential diagnosis between dementia and delusional disorder.

**Methods:**

Review of the scientific literature based on a relevant clinical case.

**Results:**

70-year-old woman who lives with her husband. She has two independent daughters. History of a depressive episode in her youth related to her husband’s gambling addiction. She attended the emergency department due to behavioural alteration at home with verbal heteroaggressiveness towards her sister and several neighbours. At the hospital she was approachable, with some psychomotor restlessness, reporting that a neighbour wanted to harm her and spoke of her, making delirious interpretations of harm and referring to visual hallucinations in the form of animals in the courtyard of her house. A brain CAT scan was performed, with normal results.

Her family reports that for about a year she has been saying incoherent things on occasions and behaving strangely. It was decided to admit her to the acute care unit.

**Conclusions:**

During hospitalisation she didn’t present behavioural alterations. Treatment with risperidone was introduced with adequate tolerance and response, with distancing of the delusional ideation of harm. MOCA test was performed: 23/30 (suggestive of cognitive impairment), so PET-CT was requested with results not suggestive of neurodegenerative disease and neurodiagnostic tests (SCIP-S and BCSE); the results indicate heterogeneous cognitive performance, and no global cognitive impairment could be observed at the present time and a repeat assessment was recommended in one year’s time. Due to the results of the tests and the decrease in positive symptomatology with antipsychotic treatment, a diagnosis of delusional disorder was made upon discharge.

**Disclosure of Interest:**

None Declared

